# Preventable hospital admissions among the homeless in California: A retrospective analysis of care for ambulatory care sensitive conditions

**DOI:** 10.1186/s12913-014-0511-7

**Published:** 2014-10-25

**Authors:** Brandi M White, Charles Ellis Jr, Kit N Simpson

**Affiliations:** Department of Health Sciences & Research, College of Health Professions, Medical University of South Carolina (MUSC), Charleston, SC USA; Department of Communication Sciences and Disorders, College of Allied Health Sciences, East Carolina University, Greenville, NC USA

**Keywords:** Homeless, Ambulatory care sensitive conditions, Primary care access

## Abstract

**Background:**

Limited research exists that investigates hospital admissions for ambulatory care sensitive conditions (ACSCs) among the homeless, who frequently lack a usual source of care. This study profiled ACSC admissions for homeless patients.

**Methods:**

Bivariate analyses and logistic regression were completed to investigate ACSC and non-ACSC admissions among homeless patients using the 2010 California State Inpatient Database.

**Results:**

Homeless patients admitted for an ACSC were mostly male, non-Hispanic white, and on average 49.9 years old. In the predictive model, the odds of an ACSC admission among homeless patients increased when they were black, admitted to the emergency department or transferred from another health facility. Having Medicare was associated with a decreased odds of an ACSC admission.

**Conclusions:**

Specific characteristics are associated with a greater likelihood of an ACSC admission. Research should examine how these characteristics contribute to ACSC hospitalizations and findings should be linked to programs designed to serve as a safety-net for homeless patients to reduce hospitalizations.

## Background

Estimates indicate that annually there are between 2.3 to 3.5 million homeless persons in the United States [1]. Homelessness is complex as many Americans find themselves homeless as a result of unemployment, debt, drug or alcohol abuse, domestic violence, mental health problems, institutionalization, and limited social support [2]. Housing concerns are compounded by challenges accessing adequate health care services and maintaining good health. Many homeless individuals experience difficulties retaining a usual source of care due to a lack of health insurance [2,3]. Consequently, many homeless individuals have worse health outcomes for preventable conditions when compared to the general population [2,4].

Many homeless persons are susceptible to unnecessary hospital admissions for ambulatory care sensitive conditions (ACSCs) [3,4]. ACSCs are defined as conditions for which good primary care would likely prevent hospitalization and reduce complications associated with the condition or more severe health outcomes as a result of the condition [5]. Hospital admissions for ACSCs are indicators of reduced or poor access to primary care services and can be used as a proxy measure for the quality of care received [6,7]. Most ACSCs are preventable or manageable with timely appropriate primary care. However, since most homeless individuals do not have a usual source of quality primary care they may be at higher risk for hospital admissions for ACSCs.

To date, little is known about ACSCs among the homeless or what factors are most associated with ACSC hospital admissions in this population. Previous studies designed to examine hospital admissions among the homeless have primarily consisted of cross-sectional, case–control or cohort studies with small sample sizes and have been limited to examinations of admissions for substance abuse and psychiatric disorders [4,8,9]. To fill this knowledge gap, this study will profile the characteristics of homeless persons admitted to hospitals in the state of California for ACSCs and identify the characteristics most predictive of a hospital admission for homeless patients being for an ACSC.

## Methods

A retrospective analysis of the 2010 State Inpatient Database (SID) for California from the Healthcare Cost and Utilization Project (HCUP) was conducted [10]. The SID provides information for 97% of all annual inpatient discharges from participating hospitals. The unit of analysis is the hospital discharge record. Clinical and nonclinical information is provided on all patients. Key variables included in the SID and used in this study were: race/ethnicity (White, Black, Hispanic, and Asian or Pacific Islander), age (18–64 years and ≥65 years; and continuous), insurance coverage (Medicaid, Medicare, private, self-pay, or other), length of stay (days), and clinical diagnoses on the discharge record. Homelessness was based on a dichotomous variable indicating whether the patient was homeless or not homeless. ACSCs were based on a dichotomous variable indicating that the primary reason for hospitalization was for an ACSC. Admission for an ACSC as the primary diagnosis was defined by Clinical Classifications Software (CCS) diagnosis categories for ICD-9-CM (*International Classification of Diseases*, *9th Revision*, *Clinical Modification*). Specific ACSCs were chosen based on previous studies and divided into chronic, acute, and preventable conditions for descriptive purposes [6,7,11]. The HCUP data used in this analysis were reviewed by the Medical University of South Carolina’s Institutional Review Board (IRB) and deemed to be non-human research that did not require additional IRB submissions.

Descriptive statistics were computed for demographic and clinical characteristics. Means and standard deviations were used to describe continuous variables; percentages were used to describe categorical variables. Independent sample t-tests or Wilcoxon tests were used to compare the means of continuous variables for an ACSC and non-ACSC admission; chi-square tests were used to determine the relationship between categorical variables. We used multivariate logistic regression to identify characteristics of the admission associated with ACSCs. Analyses were completed using SAS 9.3 (SAS Institute Inc., Cary, NC).

## Results

Approximately 4 million hospital admissions occurred in the state of California in 2010 of which 19,445 were admissions of homeless patients. Nine percent of the admissions for homeless patients were for a primary ACSC (n =1,754). The mean age of homeless patients admitted for an ACSC was 49.9 (SD [Standard Deviation] ±11.8) and admissions were mostly among men (76.8%) and non-Hispanic white patients (50.3%). On average, patients had five chronic diseases and their hospital stays were approximately five days. The average total charges for the homeless admitted for ACSCs was $45,293 (SD ± $68,930). Most patients were admitted though the emergency department (ED). Almost half of all homeless patients admitted for an ACSC had Medicare or Medicaid as a primary payer source and approximately 25% had no insurance coverage.

In comparisons between homeless patients admitted for ACSCs and non-ACSCs, those admitted for ACSCs were significantly older (49.9 vs 43.6; p < .0001) and a larger percentage were Black (23.6% vs 17.9; p < .0001). Those admitted for ACSCs had shorter lengths of stay (4.9 vs 7.9 days; p < .0001), but higher mean charges ($45,293 vs $36,935; p < .0001) when compared to those admitted for non-ACSCs. In addition, homeless patients admitted for ACSCs were more likely to be admitted through the ED and more likely to be uninsured. See Table 1 for the demographic and clinical differences between ACSC and non-ACSC admissions.

**Table 1:** **Characteristics of all admissions among the homeless admitted for a primary ACSC and non**-**primary ACSC**

**Characteristic N = ** **19,** **445**	**Primary ACSC** **(n = ** **1,** **754)**	**Non-** **primary ACSC** **(n = ** **17,** **691)**	***P-*** **value**
**Age,** **Mean** **(SD)** ^**a**^	**49.89** **(±11.8)**	**43.62** **(±12.8)**	**<.0001**
18-64 years, %	91.85%	95.36%	<.0001
≥65 years, %	7.70%	3.23%	<.0001
**Gender, %**			
Male	76.80%	74.66%	0.0487
Female	23.20%	25.34%	
**Race/** **Ethnicity, %** ^**a**^			**<.0001**
White	50.34%	51.72%	0.2703
Black	23.60%	17.92%	<.0001
Hispanic	18.64%	17.54%	0.2476
Asian or Pacific Islander	1.25%	1.18%	0.7717
Number of chronic conditions, Mean (SD)	4.81 (±2.6)	3.56 (±2.3)	<.0001
Length of stay in days, Mean (SD)	4.91 (±10.8)	7.90 (±14.5)	<.0001
Total charges in dollars, Mean (SD)	$45,293 (±68,930)	$36,935 (±73,464)	<.0001
**Admission source, %** ^**a**^			**<.0001**
Emergency Department	92.65%	48.67%	<.0001
Routine	4.68%	40.45%	<.0001
Other health facility including long-term care	1.54%	3.86%	<.0001
Another hospital	0.80%	5.03%	<.0001
**Disposition of patient, %** ^**a**^			**<.0001**
Routine	75.77%	73.18%	0.0191
Against medical advice	10.43%	8.41%	0.0039
Transfer to other	10.21%	14.89%	<.0001
Transfer to short-term hospital	1.94%	1.79%	0.6601
Home health care	1.14%	0.54%	0.0017
**Insurance coverage, %**			**<.0001**
Medicaid	36.03%	30.12%	<.0001
Self-pay	24.29%	18.51%	<.0001
Other	24.29%	31.63%	<.0001
Medicare	13.34%	15.82%	0.0064
Private	2.05%	3.83%	0.0002

^a^Percentages will not add up to 100% because of insufficient data for some categories.

*SD*: Standard deviation.

Sixty-four percent of patients admitted for ACSCs had at least one chronic condition. The most common chronic condition seen on admission was diabetes (18.93%), followed by congestive heart failure (13.97%) and chronic obstructive pulmonary disease (11.97%). Among acute conditions, pneumonia was the most common diagnosis, accounting for 14.3% of all ACSC admissions, followed by noninfectious gastroenteritis (11.23%). Admissions for preventable conditions were low and observed in only one percent of the sample (Table 2).

**Table 2:** **Admissions among the homeless admitted for an ACSC by condition type and condition**

**Ambulatory care sensitive condition**	**Percent N = ** **1,** **754**
**Chronic conditions**	**64.14**
Diabetes with/without complications	18.93
Congestive heart failure	13.97
Chronic obstructive pulmonary disease	11.97
Epilepsy/Convulsions	9.29
Asthma	5.36
Hypertension	4.62
**Acute conditions**	**34.72**
Pneumonia	14.31
Noninfectious gastroenteritis	11.23
Urinary tract infections	5.30
Gastroenteritis	1.94
Appendicitis/Other appendiceal conditions	1.43
Pelvic inflammatory disease	*NS*
**Preventable conditions**	**1.14**
Tuberculosis	0.63
Nutritional deficiencies	*NS*
Influenza	*NS*

*NS* = Data not sufficient to report.

The logistic model designed to identify predictors of a hospital admission for an ACSC, revealed that age, being non-Hispanic Black, admission through EDs and other health facilities (including long-term care), and the number of chronic conditions were predictive of an ACSC admission. With every 1-year increase in age, the odds of an admission for an ACSC increased by 2.2% (OR 1.022, 95% CI 1.017, 1.027; p < 0.0001). Similarly, for every increase in the number of chronic conditions, the odds of an admission being for an ACSC increased by 14% (OR [Odds Ratio]: 1.144; 95% CI [Confidence Interval]: 1.121, 1.168; p < 0.0001). Similarly, being non-Hispanic Black was associated with a 37% higher odds of an ACSC (OR: 1.373; 95% CI: 1.213, 1.553; p < 0.0001). Finally, homeless patients admitted through the ED had over a twelve-fold increased risk of their admission being for an ACSC (OR: 12.422; 95% CI: 10.117, 15.252, p < 0.0001) (Table 3). In contrast having Medicare was associated with a 30% lower odds of an ACSC (OR: 0.663; 95% CI: 0.566, 0.775; p < 0.0001).

**Table 3:** **Predictors for a hospital admission for an ACSC among the homeless**

**Predictor**	**Odds ratio** **(SE)**	**95%** **CI**
Age	1.022 (0.00237)	1.017, 1.027
Black or African American Race	1.373 (0.0629)	1.213, 1.553
Number of chronic conditions	1.144 (0.0104)	1.121, 1.168
Medicare insurance coverage	0.663 (0.0801)	0.566, 0.775
*Admission source*		
Emergency Department	12.422 (0.1047)	10.117, 15.252
Other health facility including long-term care	2.879 (0.2220)	1.863, 4.448

*SE*: Standard Error; *CI*: Confidence Interval.

All predictors significant at p < 0.0001.

## Discussion

The objective of this study was to profile factors associated with hospital admissions for ACSCs for the homeless using state-level (California) data. Our findings indicate that homeless adults admitted for ACSCs were significantly older, had shorter lengths of stay, and higher total hospital charges when compared to the homeless admitted for non-ACSCs. In the predictive model, the odds of an ACSC increased with age, the number of chronic conditions, and being Black. In addition, patients admitted through the ED or who were transferred from another health facility were more likely to have an ACSC as the primary diagnosis for their admission.

These findings are important for several reasons. *First*, homeless patients admitted for ACSCs were significantly older than those admitted for a non-ACSC and those admitted for ACSCs had significantly more chronic diseases than those admitted for a non-ACSC suggesting greater chronic disease burden among the patients admitted with ACSCs. Collectively, these findings are important because in the general population older persons are more susceptible to chronic illnesses and thus hospitalization. They also indicate that the chronic disease profile among older homeless adults may be similar to the general population, but exacerbated by a lack of primary care [12]. An analysis of hospitalizations among a national sample of Medicare beneficiaries found the average number of chronic conditions was 2.34, which is lower than the average for homeless persons admitted for a primary ACSC (4.81), as well as those admitted for a non-primary ACSC (3.56) [12]. It is tenable that poor living conditions, longer exposure to homelessness, and a lack of usual care may be linked to poorer health-related outcomes.

*Second*, consistent with previous research examining ACSC admissions in the general population, hospital admissions for Black patients were more likely to be for ACSCs [13,14]. ACSCs are an indicator of limited access to primary care services and serve as an indicator of racial differences in access to services, especially for high-risk populations such as the homeless [13-16]. Racial-ethnic disparities in access to preventive care have been documented extensively in the literature [15-17]. Previous studies have concluded that racial disparities in admissions for ACSCs occur as a result of social and economic factors [13,14,18]. It is possible that this disparity gap is compounded by being homeless and impacted by the social conditions in which they live.

*Third*, lengths of stay for homeless patients admitted for an ACSC were shorter compared to homeless patients admitted for non-ACSCs. However, the mean total charges associated with these shorter lengths of stays were significantly higher than those for non-ACSC admissions. These findings are interesting because length of stay is typically associated with specific diagnoses and hospital services provided. It is interesting that the homeless patients admitted for ACSCs had greater chronic disease burden which typically would contribute to a need for greater care or management and longer lengths of stays. Higher cost of care among those admitted for an ASCS suggests a higher more costly level of care, which could be associated with diagnostic tests despite shorter lengths of stays and that that shorter length of stays were associated with relatively low levels of insurance coverage in this population. A detailed study of an itemized list of hospital charges is needed to adequately answer this question.

*Fourth*, while the homeless sample reported here had a similar proportion of all admissions for ACSCs compared to the general population, the most common ACSCs observed were quite different. The most common ACSCs in previous studies were congestive heart failure, pneumonia, urinary tract infection, asthma and chronic obstructive pulmonary disease [14,18]. In contrast, ACSC admissions for diabetes, pneumonia, and congestive heart failure were the most common in this study. The finding of diabetes as the most common condition raises concerns for disease management and control. Understanding the impact of diabetes among the homeless is important because homeless persons have difficulties managing their condition because of insufficient diabetic equipment and limited access to appropriate foods [19]. Moreover, in general it is difficult for homeless persons to appropriately manage chronic conditions, thus resulting in a greater probability for ACSC hospital admissions [19].

*Fifth*, predictors of admissions for ACSCs have been quite mixed in previous studies and are likely a function of samples examined. In this sample, age, Black race, admission through EDs and other health facilities, and the number of chronic conditions predicted the presence of an ACSC among hospital admissions for homeless patients. These findings differ from Falik and colleagues who analyzed hospital admissions from Medicare and Medicaid Services and found that young age (under 14 years) and male gender predicted an ACSC admission while being white decreased the odds of being admitted [7]. Johnson and colleagues found that the odds of being admitted for an ACSC were the highest for older patients, and Black and Hispanic patients [14]. In addition, females and visits that were covered by Medicaid and Medicare were more likely to be admitted for an ACSC [14]. Interestingly, in our analysis having Medicare coverage was associated with a decreased likelihood of being admitted for an ACSC (p < .0001), which is similar to a previous study examining ACSC admissions among adult patients (≥18 years) [20].

*Sixth*, significantly more homeless patients were admitted through the ED compared to those admitted for a non-ACSC. The ED has increasingly become a healthcare safety net for those who are not insured [21]. With increased healthcare costs, unwarranted ED use is a heavy burden on the healthcare system [22]. In this database, a quarter of homeless patients admitted for an ACSC did not have health insurance, significantly more than those admitted for a non-ACSC. A previous analysis of the SID found that almost three out of four homeless patients were admitted through the ED, almost half of which were uninsured [23]. Many conditions that homeless patients are admitted for are preventable, indicating possible gaps in the healthcare safety net for the homeless [9]. In essence, the ED is the safety net for many homeless because of limited access to primary healthcare services and/or inadequate healthcare coverage [9,24-26].

The findings reported here are interesting but are not without limitations. First, it is unclear how homelessness was identified. It is possible that reporting may have been increased or decreased based on patient motivation to receive services or patient refusal to acknowledge homelessness because of the potential stigmatization associated with reporting. Second, readmission rates were not available in the 2010 California SID and thus not accounted for. Third, these findings may not be generalized to homeless populations in other states due to variable availability of social services. Fourth, we did not compare hospital admissions for ASCSs with the non-homeless population. Future research warrants further investigations to compare admissions between the homeless and general populations to identify healthcare service needs. Despite the limitations, these findings fill a gap in the literature examining hospital admissions for ACSCs for the homeless.

Despite these limitations, the findings reported here are important because increased insurance coverage for homeless persons could potentially improve access to preventive services, reduce ED admissions for ACSCs, and potentially improve overall health outcomes [27]. The homeless patients with Medicare in this sample were less likely to be admitted for an ACSC, indicating that these patients may have a regular source of primary care because of insurance coverage, thus reducing their admission for ACSCs. To increase healthcare coverage, the Health Resources and Services Administration (HRSA) has offered funding for healthcare centers for the homeless. The HRSA programs allocate funds to fund clinics focused on providing care to homeless persons [28]. While only half of patients visiting these centers have healthcare insurance, the centers address the needs of the homeless including transportation and assistance with medication management, to increase access and improve health outcomes [29]. In addition, funding from the Patient Protection and Affordable Care Act (ACA) which expands Medicaid coverage may offer relief for the homeless [30] This expansion could potentially increase healthcare coverage to homeless adults since many homeless persons did not previously qualify. For states that participate in the expansion, homeless persons could have an opportunity to obtain health insurance, which would increase access to primary care services. However, any effort to increase healthcare insurance in this population must increase outreach efforts and provide targeted assistance to increase enrollment [29].

## Conclusions

This study demonstrated that homeless patients admitted for an ACSC rely heavily on EDs for healthcare services. These are conditions that are manageable with timely and appropriate primary care. This study is one of a few to use state-level hospital discharge data to characterize hospital admissions for preventable conditions among homeless patients and is the first step in understanding factors associated with preventable hospital admissions among homeless patients. Homelessness is costly to society and the healthcare system. While affordable and stable housing is the key solution to prevent homelessness, in the interim, healthcare and public health practitioners must identify strategies to improve health outcomes in this population. Improving access to primary healthcare services and increasing insurance coverage for the homeless are effective approaches to reduce healthcare costs. These findings can be used to tailor clinical and public health interventions for the homeless to reduce admissions for preventable conditions.

## Abbreviations

AHRQ: Agency for Healthcare Research and Quality
ACSCs: Ambulatory care sensitive conditions
ACA: Patient Protection and Affordable Care Act
CCS: Clinical Classifications Software
ED: Emergency department
HRS: Health Resources and Services Administration
HCUP: Healthcare Cost and Utilization Project
SID: State Inpatient Database

## References

[1] Burt MR, Aron LY, Lee E, Valent J (2001). How many homeless people are there?: Helping America’s Homeless: Emergency Shelter or Affordable Housing?.

[2] Wright NM, Tompkins CN (2006). How can health services effectively meet the health needs of homeless people?. Br J Gen Pract.

[3] Kushel MB, Vittinghoff E, Haas JS (2001). Factors associated with the health care utilization of homeless persons. JAMA.

[4] Hwang SW, Orav EJ, O’Connell JJ, Lebow JM, Brennan TA (1997). Causes of death in homeless adults in Boston. Ann Intern Med.

[5] AHRQ (2001). AHRQ Quality Indicators—Guide to Prevention Quality Indicators: Hospital Admission for Ambulatory Care Sensitive Conditions.

[6] Ansari Z, Haider SI, Ansari H, De Gooyer T, Sindall C (2012). Patient characteristics associated with hospitalisations for ambulatory care sensitive conditions in Victoria, Australia. BMC Health Serv Res.

[7] Falik M, Needleman J, Wells BL, Korb J (2001). Ambulatory care sensitive hospitalizations and emergency visits: Experiences of Medicaid patients using federally qualified health centers. Med Care.

[8] Adams J, Rosenheck R, Gee L, Seibyl CL, Kushel M (2007). Hospitalized younger: A comparison of a national sample of homeless and housed inpatient veterans. J Health Care Poor Underserved.

[9] Salit SA, Kuhn EM, Hartz AJ, Vu JM, Mosso AL (1998). Hospitalization costs associated with homelessness in New York City. N Engl J Med.

[10] Healthcare Cost and Utilization: *Overview of the State Inpatient Databases.* [http://www.hcup-us.ahrq.gov/sidoverview.jsp]

[11] Purdy S, Griffin T, Salisbury C, Sharp D (2009). Ambulatory care sensitive conditions: terminology and disease coding need to be more specific to aid policy makers and clinicians. Public Health.

[12] Wolff JL, Starfield B, Anderson G (2002). Prevalence, expenditures, and complications of multiple chronic conditions in the elderly. Arch Intern Med.

[13] Burr J, Sherman G, Prentice D, Hill C, Fraser V, Kollef MH (2003). Ambulatory care-sensitive conditions: Clinical outcomes and impact on intensive care unit resource use. South Med J.

[14] Johnson PJ, Ghildayal N, Ward AC, Westgard BC, Boland LL, Hokanson JS (2012). Disparities in potentially avoidable emergency department (ED) care: ED visits for ambulatory care sensitive conditions. Med Care.

[15] Mayberry RM, Mili F, Ofili E (2000). Racial and ethnic differences in access to medical care. Med Care Res Rev.

[16] Gaskin DJ, Hoffman C (2000). Racial and ethnic differences in preventable hospitalizations across 10 states. Med Care Res Rev.

[17] Sambamoorthi U, McAlpine DD (2003). Racial, ethnic, socioeconomic, and access disparities in the use of preventive services among women. Prev Med.

[18] Howard DL, Hakeem FB, Njue C, Carey T, Jallah Y (2007). Racially disproportionate admission rates for ambulatory care sensitive conditions in North Carolina. Public Health Rep.

[19] Hwang SW, Bugeja AL (2000). Barriers to appropriate diabetes management among homeless people in Toronto. CMAJ.

[20] Chang CF, Troyer JL (2011). Trends in potentially avoidable hospitalizations among adults in Tennessee, 1998–2006. Tenn Med.

[21] Tang N, Stein J, Hsia RY, Maselli JH, Gonzales R (2010). Trends and characteristics of US emergency department visits, 1997–2007. JAMA.

[22] Caldwell N, Srebotnjak T, Wang T, Hsia R (2013). “How Much Will I Get Charged for This?” Patient Charges for Top Ten Diagnoses in the Emergency Department. PLoS One.

[23] Karaca Z, Wong H, Mutter R (2013). Characteristics of Homeless and Non-Homeless Individuals Using Inpatient and Emergency Department Services, 2008.

[24] Kushel MB, Perry S, Bangsberg D, Clark R, Moss AR (2002). Emergency department use among the homeless and marginally housed: results from a community-based study. Am J Public Health.

[25] D’Amore J, Hung O, Chiang W, Goldfrank L (2001). The epidemiology of the homeless population and its impact on an urban emergency department. Acad Emerg Med.

[26] Gallagher TC, Andersen RM, Koegel P, Gelberg L (1997). Determinants of regular source of care among homeless adults in Los Angeles. Med Care.

[27] Stein JA, Andersen RM, Koegel P, Gelberg L (2000). Predicting health services utilization among homeless adults: a prospective analysis. J Health Care Poor Underserved.

[28] Bureau of Primary Health Care, Health Resources and Services Administration (2008). Health Centers: America’s Primary Care Safety Net, Reflections on Success, 2002–2007.

[29] Lebrun-Harris LA, Baggett TP, Jenkins DM, Sripipatana A, Sharma R, Hayashi AS, Daly CA, Ngo-Metzger Q (2013). Health Status and Health Care Experiences among Homeless Patients in Federally Supported Health Centers: Findings from the 2009 Patient Survey. Health Serv Res.

[30] Tsai J, Rosenheck RA, Culhane DP, Artiga S (2013). Medicaid expansion: Chronically homeless adults will need targeted enrollment and access to a broad range of services. Health Aff (Millwood).

